# Bone transport through an induced membrane in the management of tibial bone defects resulting from chronic osteomyelitis

**DOI:** 10.1007/s11751-015-0221-7

**Published:** 2015-04-04

**Authors:** Leonard Charles Marais, Nando Ferreira

**Affiliations:** Tumor, Sepsis and Reconstruction Unit, Department of Orthopaedic Surgery, Greys Hospital, University of KwaZulu-Natal, Private bag X9001, Pietermaritzburg, 3201 South Africa

**Keywords:** Chronic osteomyelitis, Bone transport, Distraction osteogenesis, Induced membrane, Masquelet technique, Circular external fixation

## Abstract

Wide resection of infected bone improves the odds of achieving remission of infection in patients with chronic osteomyelitis. Aggressive debridement is followed by the creation of large bone defects. The use of antibiotic-impregnated PMMA spacers, as a customized dead space management tool, has grown in popularity. In addition to certain biological advantages, the spacer offers a therapeutic benefit by serving as a vehicle for delivery of local adjuvant antibiotics. In this study, we investigate the efficacy of physician-directed antibiotic-impregnated PMMA spacers in achieving remission of chronic tibial osteomyelitis. This retrospective case series involves eight patients with chronic osteomyelitis of the tibial diaphysis managed with bone transport through an induced membrane using circular external fixation. All patients were treated according to a standardized treatment protocol. A review of the anatomical nature of the disease, the physiological status of the host and the outcome of treatment in terms of remission of infection, time to union and the complications that occurred was carried out. Seven patients, with a mean bone defect of 7 cm (range 5–8 cm), were included in the study. At a mean follow-up of 28 months (range 18–45 months), clinical eradication of osteomyelitis was achieved in all patients without the need for further reoperation. The mean total external fixation time was 77 weeks (range 52–104 weeks), which equated to a mean external fixation index of 81 days/cm (range 45–107). Failure of the skeletal reconstruction occurred in one patient who was not prepared to continue with further reconstructive surgery and requested amputation. Four major and four minor complications occurred. The temporary insertion of antibiotic-impregnated PMMA appears to be a useful dead space management technique in the treatment of post-infective tibial bone defects. Although the technique does not appear to offer an advantage in terms of the external fixation index, it may serve as a useful adjunct in order to achieve resolution of infection.

## Introduction

Wide resection of infected bone improves the odds of relapse-free periods in patients with chronic osteomyelitis. Aggressive debridement creates segmental bone defects. While small defects may be managed with acute shortening or cancellous bone grafting, larger segmental bone defects typically require bone transport with regeneration of the deficient bone segment through distraction osteogenesis [1]. The size of critical bone defect which by definition cannot be managed with cancellous bone graft remains controversial. Tiemann et al. [2] recommended 2 cm as the maximum size of a segmental diaphyseal tibial defect that should be managed with autologous cancellous grafting alone.

The induced membrane (Masquelet) technique, involving the placement of a polymethylmethacrylate (PMMA) spacer in the defect with subsequent bone grafting, has emerged as a useful adjunct in the management of large defects. The induced membrane is highly vascularized and secretes several growth factors, including VEGF and BMP-2 [3]. Furthermore, extracts from the membrane have been shown to stimulate bone marrow cell proliferation and differentiation of progenitor cells to the osteoblast lineage [4]. These factors combine to facilitate successful consolidation of cancellous bone graft within an induced membrane in segmental tibial defects of up to 25 cm in length [5].

Distraction osteogenesis remains a method of choice for the management of bone defects in excess of 4 cm [6, 7]. The procedure offers several advantages in the scenario of post-osteomyelitis skeletal reconstruction, including the increase in regional blood flow for a period up to 17 weeks following the corticotomy [8]. Although bone transport can be achieved with various devices, circular external fixation in accordance with Ilizarov principles remains foremost due to its reliability, modularity and safety in the presence of infection.

The use of antibiotic-impregnated PMMA spacers, as a customized dead space management tool after debridement for chronic osteomyelitis, has grown in popularity [9, 10]. Apart from the biological advantages illustrated by Masquelet et al., the spacer offers potential therapeutic benefit as a vehicle for delivery of local adjuvant antibiotics. Physician-directed antibiotic-impregnated PMMA spacers have been shown to effectively elute antibiotics at the site of infection for up to several months following implantation [11]. This characteristic has been used to good effect in periprosthetic infections where staged reconstruction has been shown to be safe after removal of the spacer [12]. The biological, mechanical and therapeutic advantages offered by the Masquelet technique have prompted the use of antibiotic-impregnated PMMA spacers in larger segmental defects requiring bone transport.

The aim of this study was to determine whether the use of antibiotic-impregnated PMMA spacers followed by bone transport with circular external fixation is effective in achieving remission of infection following segmental resection in chronic tibial osteomyelitis. A secondary objective was to determine the external fixation index of these cases and to compare it to those of other authors using traditional Ilizarov methods.

## Materials and methods

A retrospective review was conducted of all patients treated by bone transport through an induced membrane at our tertiary level limb reconstruction unit over a 4-year period between June 2009 and June 2013. All adult patients treated for a diaphyseal tibial bone defect by bone transport were included in the study. Patients were excluded if the standard treatment protocol was not completed. The subjects’ charts were reviewed and data extracted in order to describe the patient demographics, cause of the bone defect, physiological status of the host in accordance with the Cierny and Mader classification system, relevant local and systemic risk factors, the number and nature of surgical procedures performed, time to union and, finally, the complications that occurred.

**Fig. 1:**
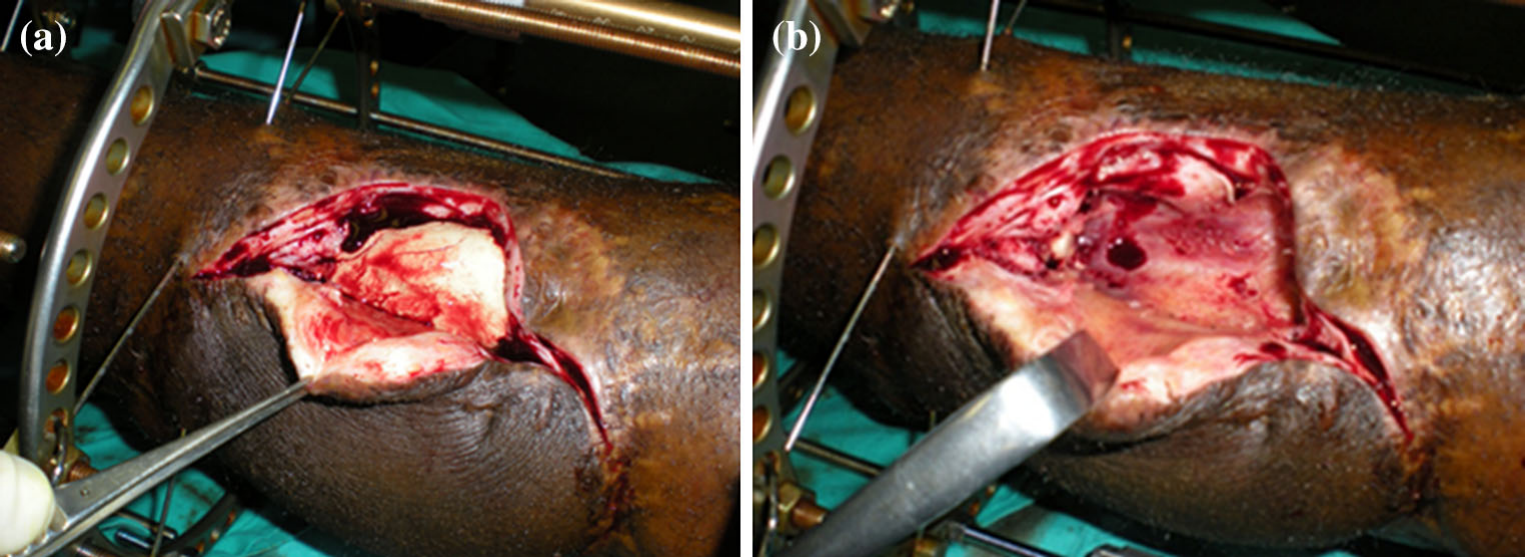
**a** Antibiotic-impregnated PMMA spacer, which was inserted into the bone defect prior to soft tissue cover and stabilization. **b** Induced membrane at time of removal of the spacer

All patients were treated according to a standardized treatment protocol. Following comprehensive clinical, biochemical and radiological evaluation, patients were classified according to the Cierny and Mader classification system [13]. The initial surgical procedure included a wide resection of all necrotic and ischemic tissue to a well-perfused margin, insertion of an antibiotic-impregnated PMMA spacers in the resulting bone defect, reconstruction of the soft tissue defect with a local flap and application of a standard five ring circular fine wire external fixator capable of effecting bone transport (Fig. 1). The PMMA spacers were constructed from Palacos R+G^®^ bone cement (Heraeus Medical, Hanau, Germany) containing 500 mg gentamicin per 40 mg of PMMA powder, mixed with 2 g of vancomycin powder per 40 mg of PMMA. In the majority of cases, the spacer was shaped outside the body and only inserted into the defect once it had hardened and most of the heat had dissipated. In later cases, the PMMA spacer was inserted before the cement had completely hardened, in order to allow the ends of the cement to overlap the bone ends.

Post-operatively, all patients were treated with generic parenteral antibiotics, in the form of vancomycin and meropenem, until results from 7-day microscopy, cultures and microbial antibiotic sensitivity (MCS) became available. Oral antibiotic therapy, tailored according to the results of culture and sensitivity, was then commenced and continued for a period of 6 weeks. During this initial period, the patient was allowed to mobilize partial weight-bearing in order to curtail disuse osteopenia. The second-stage procedure was performed after a minimum of 6 weeks from the index procedure and only if there was no clinical or biochemical evidence of ongoing infection as indicated by normal white blood cell count, C-reactive protein and erythrocyte sedimentation rate. The second-stage procedure involved removal of the spacer through an incision at the edge of the flap, debridement of the bone edges, as well as suturing of the incision made in the induced membrane. At the same sitting, a metaphyseal osteotomy was performed, according to the technique described by De Bastiani, in preparation for bone transport [14]. A latency period of 7 days was observed prior to commencement of bone transport which was performed according to standard Ilizarov principles of 0.25 mm distraction increments, four times per day [15]. During this stage of the treatment protocol, full weight-bearing, with no more than a single crutch, was advocated.

Once the bone ends were brought into close apposition through bone transport, a formal docking procedure was performed in the form of a cancellous and Phemister-type bone graft [16]. No internal fixation of docking sites or regenerated segments was performed. Pin track care was performed according to a previously published protocol [17]. When three out of the four cortices of the regenerate were judged well formed on AP and lateral X-rays and the docking site had united, the circular fixator was removed. The external fixation index was defined as the total time of external fixation per centimetre of bone transport.

Ethical approval was obtained from the relevant ethics review board prior to commencement, and the study was performed in accordance with the pertinent ethical guidelines.

## Results

The records of eight patients, who were referred to our unit with Cierny and Mader anatomical type IV chronic osteomyelitis of the tibial diaphysis, were reviewed. One patient, who did not complete the standard treatment protocol, was excluded from the study. The follow-up period for this case was <18 months. Of the remaining seven patients, chronic osteomyelitis occurred after treatment for open tibia fractures in six and the final patient developed contiguous osteomyelitis following an open reduction and intramedullary nail for failed non-operative management of a closed fracture of the tibia shaft.

The mean age of patients was 29 years (range 28–44 years), and the mean time from injury to referral to our unit was 3 months (ranging from 1 to 14 months). Systemic risk factors, namely hypoalbuminemia, substance-induced psychiatric disorder and cigarette smoking, were identified in five of the patients (Table 1). The mean follow-up period in this series of patients was 28 months (range 18–45 months). The mean interval between the index (first stage) procedure and removal of the spacer and tibial osteotomy (second stage) was 12 weeks (range 9–28 weeks), and the mean time from the second-stage procedure to bone grafting of the docking site was 17 weeks (8–39 weeks).

**Table 1:** Risk factors, magnitude of bone defect, treatment intervals, follow-up duration and complications

Patient	Age	Systemic risk factors	Post-debridement bone defect (cm)	First to second stage (weeks)	Time in frame (weeks)	Fixator index (days/cm)	Follow-up period (months)	Complications
1	30	Substance-induced psychotic disorder	8	14	52	45	28	Knee flexion and ankle equinus contractures requiring unplanned reoperation
2	28	Hypoalbuminemia	5	28	77	107	30	Flap dehiscence, fracture of docking site requiring second external fixator
3	28	Smoking	7	11	104	104	45	Pin track sepsis necessitating removal of one wire (Checketts and Otterburn grade 3), 5° equinus contracture
4	39	None	8	13	80	70	41	None
5	29	None	6	12	58	67	21	New circular fixator with acute compression of docking site at formal docking
6	44	Poor compliance, smoking	8	n/a	n/a	n/a	21	Flap dehiscence, pin track sepsis (Checketts and Otterburn grade 2), patient eventually requested amputation
7	30	Smoking	5	9	58	81	18	Pin track sepsis (Checketts and Otterburn grade 2)

Clinical resolution of infection was achieved in all patients as indicated by normal clinical and biochemical findings at last follow-up. The mean magnitude of the bone defect following debridement was 7 cm (range 5–8 cm). Leg length was restored to within 1 cm of the contralateral side in all of the cases. Union of the docking site and consolidation of the regenerated segment was achieved in all but one of the cases. This patient was poorly compliant with the follow-up, rehabilitation and circular fixator care programs; he requested an amputation 17 months after presentation. The median value of the total time spent in the circular external fixator was 77 weeks (ranging from 52 to 104 weeks), and the mean external fixation index was 81 days/cm (range 45–107).

**Fig. 2:**
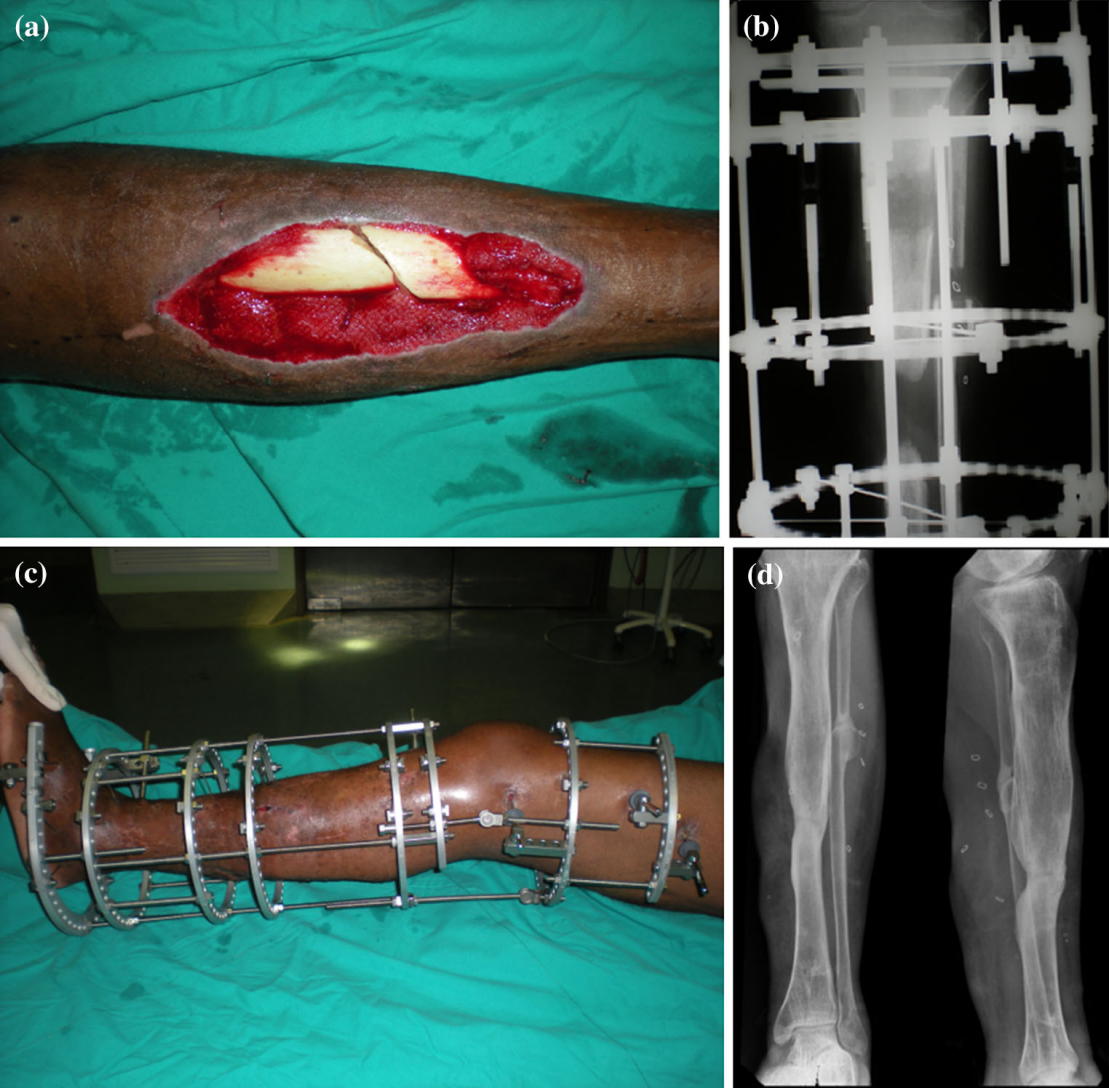
Clinical and radiological features of a case complicated by knee flexion and equinus contractures. **a** Wound dehiscence following open reduction and intramedullary nailing of a neglected tibia fracture. **b** Distraction osteogenesis following removal of the PMMA spacer. **c** Gradual correction of the knee and ankle deformities. **d** Final radiographs showing satisfactory consolidation of the regenerate and union at docking site

Complications were common and occurred in six of the seven cases. Unplanned additional surgeries were required in two patients, and the circular external fixator of one patient was revised at the time of the formal docking procedure in order to create the optimal biomechanical environment for union at the docking site (Table 1). Four major complications occurred. Despite the fact that all soft tissue flaps were performed by a plastic surgeon, flap dehiscence occurred in two cases. A flexion contracture of the knee combined with an equinus contracture of the ankle occurred in one patient (who had an associated substance-induced psychotic disorder). These deformities necessitated extension of the circular external fixator frame across the knee and ankle joints to allow gradual correction (Fig. 2). In one patient, a fracture of the docking site occurred 1 year following removal of the circular fixator. This fracture was treated successfully with a second circular fixator combined with a fibula osteotomy; union occurred after 21 weeks in external fixation.

Four minor complications occurred. A functional range of motion of the adjacent joints was achieved in all but one patient with a residual equinus contracture of five degrees. Minor pin track infection (Checketts and Otterburn grade 2 and 3) was experienced in three cases and necessitated the removal of the offending wire in one of these patients [18]. The incidence of pin track sepsis did not appear to differ from a previous study involving circular fixation [19].

## Discussion

Surgical resection of avascular bone should be considered a mainstay of treatment when embarking on a curative treatment strategy aimed at eradication of infection in patients with chronic osteomyelitis [6]. Systemic and local antibiotic therapy is considered to play an adjunctive role in this setting. The resection margin, which can be thought of in oncological terms as being either marginal or wide, has been shown to affect the outcome. Wide resection margins, in comparison with marginal resection, have been shown to decrease the recurrence of infection and improve cure rates [20–22]. Wide resection of all avascular bone creates large bone defects and necessitates the implementation of an appropriate dead space management strategy [23].

The management of post-infective bone defects is dependent on several factors including the host’s physiological status, the size of the defect, duration of the defect (i.e. acute or chronic), quality of the surrounding soft tissue, the presence of deformity, joint contracture/instability or limb length discrepancy, as well as the experience of the surgeon. Smaller defects may be treated by autologous bone graft [24]. The size of a segmental bone defect that should be considered critical, and thus not suitable for autologous cancellous bone grafting, remains controversial. Traditionally, 4 cm has been recommended as the cut-off point [1, 25]. The main concern with cancellous bone grafting of larger bone defects is its dependence on the surrounding soft tissues for incorporation. Large grafts may undergo central necrosis in the absence of an excellent soft tissue envelope [2]. Secondly, the regenerated segment may be weak and prone to fracture as a result of partial graft resorption [26]. As a result, it has been recommended that the length of a segmental diaphyseal tibial defect that can be managed with autologous cancellous grafting should not exceed 2 cm [14].

Masquelet et al. redefined the role of cancellous bone grafting in limb reconstruction by taking advantage of the mechanical and biological characteristics of the induced membrane. They reported the successful use of this technique in 35 cases, with defects ranging from 4 to 25 cm [3]. These results appear to be reproducible, with others reporting 90 % union rates of large defects (average size 5.8 cm) through the use of reamer–irrigation–aspiration graft [27]. Richards et al. [28] also utilized a modification of this technique in the management of bone loss following open fractures. Through the use of form-fitting spacers and subsequent autogenous bone grafting, the authors were able to achieve union in 18 out of 18 patients with bone defects involving a minimum of 50 % of the circumference of the tibia, ranging in size from 2 to 16 cm (average 4 cm).

Although the induced membrane technique offers several theoretical and practical advantages, caution should be applied in the use of cancellous bone graft in defects exceeding 4 cm [14]. Furthermore, the classic Masquelet technique, involving cancellous grafting onto the induced membrane, appears to deliver more predictable results in the presence of pre-existing periosteal new bone formation at the margins of the defect. In their original series, Masquelet et al. [3] reported that some of the patients required repeated bone grafts and that fracture occurred in four of the 35 cases. In their subsequent, prospective series that involved the adjunctive use of BMP-7, three of the eight patients with segmental defects developed deformities and another patient required amputation. Although Stafford et al. reported a high union rate, 80 % of their patients received BMP in addition to bone graft, only nail or plate fixation was used and seventeen of the 25 defects were ≤4 cm in size [18]. Richards et al. reported excellent results with the use of form-fitting spacers in the management of post-traumatic bone loss, but only two of their eighteen patients had circumferential bone loss and all fractures were treated with nail and plate fixation.

PMMA spacers offer several potential advantages in the setting of post-infective reconstruction. Bone transport through scar tissue, using more traditional Ilizarov techniques, can be particularly problematic. The use of temporary PMMA spacers prevents soft tissue impingement between the leading edge of the transport segment and the target segment. As illustrated in an animal model, the induced membrane prevents protrusion of adjacent soft tissue and neurovascular structures into the defect and adheres to the resected bone edges without collapse despite removal of the spacer, thus delineating a cavity corresponding to the volume of the retrieved cement spacer [6, 29]. This so-called spacer effect can be utilized in the reconstruction of bone defects where the resulting cylindrical cavity forms a stable envelope through which a bone segment may be transported.

As a result of the biological, therapeutic and mechanical advantages offered, and supported by the positive results reported in periprosthetic infections, antibiotic-impregnated PMMA spacers appear to be an attractive option in the management of other Cierny and Mader anatomical type IV infections associated with a bone defect. A case report where a similar technique, involving the use of a PMMA spacer with subsequent distraction osteogenesis, was used in the management of an infected open fracture is published [10]. The authors made use of a monolateral external fixator for bone transport. Circular external fixation was preferred in our series due to its modularity, minimally invasive nature and ability to effect bone transport and deformity correction (as illustrated in the case which developed joint contractures). The attributes of fine wire external fixators may also offer theoretical advantages in terms of bone healing. This stems from the three-dimensional stability combined with the low axial stiffness exhibited by fine wire circular external fixators [30, 31]. In addition, meta-analysis has shown that the Ilizarov method of distraction osteogenesis significantly reduces the risk of deep infection in infected osseous lesions [32].

Spiegl et al. [9] have published a series of cases where segmental bone defects as a result of chronic tibial osteitis were managed with PMMA spacers and subsequent distraction osteogenesis. The authors report an average overall treatment time of 93 weeks. Complications were common, and infection requiring reoperation occurred in 28 % of cases at 2-year follow-up. In our series of cases, the technique of bone transport through an induced membrane was confirmed to be a useful option for reconstruction of post-infective tibial defects in excess of 4 cm. Remission of infection was achieved in all cases without the need for reoperation for infection. This is, however, also a function of patient selection, and only Cierny and Mader type A and B hosts were considered suitable candidates for this procedure. The improved cure rates in our series may be the result of judicious patient selection rather than technical prowess. Union of the docking site and consolidation of the regenerated segment was achieved in all but one of the cases (who elected to have an amputation). Traditional Ilizarov methods, involving monofocal strategies without spacers, have been noted to produce external fixation indices of up to 50 days/cm [33, 34]. Considering the high external fixation index in our series (mean 81 days/cm), as well as the 57 days/cm reported by Speigl et al. [9], it appears that the use of PMMA spacers necessitate an increased period of external fixation. This may partly be explained by the time spent awaiting the formation of the induced membrane prior to initiation of bone transport.

There was a high rate of complications in our series. Spiegl et al. [9] had a similar experience with this technique, reporting 22 minor and 13 major complications (including one amputation) in their series of 19 cases. Interestingly, internal fixation of the docking site was performed in 16 of their 25 patients. The authors emphasized the challenging nature of the technique and stated that the procedure places considerable physical and emotional stress on the patient. The frequency of complications may be a reflection of the complexity of the cases involved but may also be related to the technical demands of the procedure.

There are several limitations to this study: its retrospective nature; a small sample size and short follow-up period; as well as the lack of a control group involving traditional Ilizarov-type bone transport. The Masquelet technique is still relatively new and many questions remain. Further investigation is required regarding the possibility of improved union at the docking site related to the biological advantages offered by the induced membrane. This will require a control group of cases managed without PMMA spacers. Our knowledge of certain technical aspects is still evolving. The optimal time for removal of the spacer and initiation of bone transport remains unclear. Internal fixation of the docking site may possibly also offer additional benefit [9].

## Conclusion

The temporary insertion of a PMMA appears to be a useful dead space management technique in the treatment of post-infective tibial bone defects. Although the technique does not appear to offer an advantage in terms of the external fixation index, it may serve as a useful adjunct in achieving resolution of infection. Patient selection, however, appears to be a crucial step in ensuring a remission of tibial osteomyelitis.
